# Artificial Intelligence-Driven Diagnostic Processes and Comprehensive Multimodal Models in Pain Medicine

**DOI:** 10.3390/jpm14090983

**Published:** 2024-09-16

**Authors:** Marco Cascella, Matteo L. G. Leoni, Mohammed Naveed Shariff, Giustino Varrassi

**Affiliations:** 1 Anesthesia and Pain Medicine, Department of Medicine, Surgery and Dentistry “Scuola Medica Salernitana”, University of Salerno, 84081 Baronissi, Italy; mcascella@unisa.it; 2 Department of Medical and Surgical Sciences and Translational Medicine, Sapienza University of Roma, 00185 Rome, Italy; 3 Department of AI&DS, Rajalakshmi Institute of Technology, Chennai 600124, Tamil Nadu, India; 4 Fondazione Paolo Procacci, 00193 Rome, Italy

**Keywords:** artificial intelligence, pain, automatic pain assessment, pain diagnosis

## Abstract

Pain diagnosis remains a challenging task due to its subjective nature, the variability in pain expression among individuals, and the difficult assessment of the underlying biopsychosocial factors. In this complex scenario, artificial intelligence (AI) can offer the potential to enhance diagnostic accuracy, predict treatment outcomes, and personalize pain management strategies. This review aims to dissect the current literature on computer-aided diagnosis methods. It also discusses how AI-driven diagnostic strategies can be integrated into multimodal models that combine various data sources, such as facial expression analysis, neuroimaging, and physiological signals, with advanced AI techniques. Despite the significant advancements in AI technology, its widespread adoption in clinical settings faces crucial challenges. The main issues are ethical considerations related to patient privacy, biases, and the lack of reliability and generalizability. Furthermore, there is a need for high-quality real-world validation and the development of standardized protocols and policies to guide the implementation of these technologies in diverse clinical settings.

## 1. Introduction

Pain diagnosis is often highly challenging due to several factors. These barriers include, but are not limited to, the inherently subjective nature of pain, the wide range of pain expressions that are not always clearly distinguishable, and the complexity of identifying a symptom that is intertwined with broader biopsychosocial factors [1,2].

In this complex scenario, the potential assistance from technology becomes increasingly relevant. Specifically, artificial intelligence (AI) is emerging as a powerful tool in medicine and healthcare [3]. It is a multidisciplinary research field involving the use of mathematical approaches, statistics, and advanced algorithms to simulate human decision-making and problem-solving [4].

The integration of AI into pain medicine has become a significant area of research [5]. Early developments centered around data management and basic predictive models, showcasing the potential of computational techniques in diagnosing pain conditions, such as low back pain [6] and abdominal pain [7]. With the emergence of big data and more advanced machine learning (ML) techniques in the 2010s, AI’s applications expanded to include more complex tasks, like image analysis and sophisticated predictive analytics, marking the beginning of a new era in precision pain medicine across various clinical settings [8]. Given that ML algorithms and deep learning (DL) can process complex datasets to identify patterns and make predictions, AI has been applied in pain medicine for a range of purposes. In the area of computer-aided diagnosis (CAD), efforts have been made to improve diagnostic accuracy for pain [9], predict treatment outcomes [10], and tailor pain management strategies [11]. Moreover, generative AI, particularly natural language processing (NLP), has been used to extract valuable information from clinical notes and patient reports, contributing to the development of specialized chatbots [12]. Additionally, automated tools such as smartphone apps and wearable devices have been designed to provide real-time monitoring and analysis of pain-related data [13]. These technologies enable the integration of various data sources, including self-reported pain levels, facial expressions, and behavioral and physiological signals [13]. This review focuses on computer-aided pain diagnosis, particularly on the role of AI within comprehensive multimodal models. Limitations, ethical concerns, and perspectives are also addressed.

## 2. Computer-Aided Diagnosis

### 2.1. Automatic Pain Assessment

AI-driven diagnostic methods hold significant promise, particularly in complex cases where the cause of pain is multifactorial and not easily identifiable [14]. The use of multimodal approaches is considered beneficial for delivering a more precise and comprehensive pain assessment compared to relying solely on subjective self-reports. Therefore, automatic pain assessment (APA) is a set of research and clinical approaches designed to offer objective and quantifiable measures of pain, aiming to reduce dependence on subjective self-reports. 

**Figure 1:**
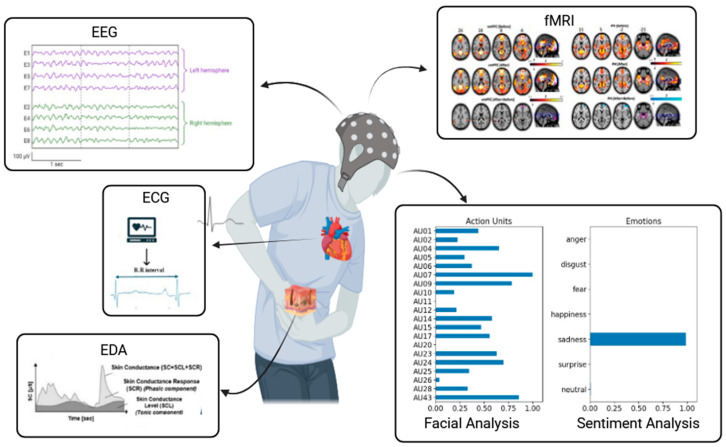
Approaches for automatic pain assessment. EDA, electrodermal activity; EEG, electroencephalography; ECG, electrocardiography; fMRI, functional magnetic resonance imaging (fMRI).

APA methodologies generally fall into two primary categories: (1) behavioral-based approaches, which involve analyzing facial expressions, linguistic cues, and nonverbal physical indicators such as body movements, and (2) neurophysiology-based pain detection methods. The latter group includes biosignal strategies such as electrodermal activity (EDA), electroencephalography (EEG), electrocardiography (ECG), electromyography (EMG), and photoplethysmography (PPG), as well as imaging methods such as functional magnetic resonance imaging (fMRI) and other approaches [13] (Figure 1).

Given their properties, AI-driven APA systems have the potential to revolutionize pain management. Specifically, by training models to detect patterns and correlations that may not be evident to human observers, these systems can identify subtle indicators of pain that might be otherwise unnoticed. Additionally, AI-driven APA systems benefit from iterative learning processes, allowing them to continually enhance their performance. This feature can also allow them to incorporate feedback from clinical outcomes and patient reports. This adaptability is particularly significant in pain medicine. For instance, in cancer patients, breakthrough pain—a sudden and severe flare-up that occurs despite otherwise well-controlled pain—is a challenging phenomenon [15]. AI-based APA models could be developed to anticipate or detect these distressing symptoms, offering timely interventions. Furthermore, AI’s ability to integrate multimodal data enables the creation of a comprehensive view of a patient’s pain experience by combining information from various sources. Unsupervised ML models, for example, can uncover clusters by merging structured and unstructured data, providing insights into pain trajectories, the impact of pharmacological and non-pharmacological therapies, and critical issues like opioid prescriptions and misuse.

This holistic approach not only enhances the accuracy of pain assessments but also supports the development of personalized pain management strategies. Since the National Institutes of Health (NIH) Biomarkers Definitions Working Group defined a biomarker as “a characteristic that is objectively measured and evaluated as an indicator of normal biological processes, pathogenic processes, or pharmacologic responses to a therapeutic intervention” [16], the ambitious goal of AI-based APA strategies is to offer pain biomarkers useful in different scenarios of pain medicine.

### 2.2. AI Strategies Implemented

Various CAD strategies have been developed to support clinicians in achieving more accurate diagnoses. AI models are developed by implementing different ML algorithms and deep learning artificial neural networks (ANNs). The latter are computational models inspired by the human brain’s network of neurons. Briefly, they consist of interconnected nodes or “neurons” organized in layers, comprising an input layer, one or more hidden layers, and an output layer. These approaches are used to recognize patterns, make predictions, and solve complex problems. Therefore, ANNs are commonly applied in tasks such as image and speech recognition, language processing, and predictive analytics. ANNs have also been used for APA research. For example, Fontaine et al. [17] demonstrated that their deep learning model, a ResNet-18 convolutional neural network, which analyzed 2810 facial expressions from 1189 patients before and after surgery, achieved a sensitivity of 89.7% for detecting pain, 77.5% for severe pain, and an accuracy of 53% in estimating pain intensity. In contrast, Bargshady et al. [18] utilized a three-stream hybrid deep neural network (ensemble deep learning model) trained on a pain database, reporting an accuracy of 89% and an AUC ROC of 94% for shoulder pain estimation. Similarly, Hosseini et al. [19] employed a deep learning model with transfer learning, utilizing a pre-trained CNN model with modified upper layers to identify seven pain intensity levels from facial expressions. Additionally, Barua et al. [20] used a pre-trained DarkNet19 network to achieve a high accuracy of 95% in estimating self-reported shoulder pain. These studies primarily focused on facial expressions for pain recognition.

### 2.3. Diagnosis of Low Back Pain

Other innovative approaches have been explored, particularly for low back pain. Abdollahi et al. [21] conducted a study using an inertial measurement unit (IMU) to gather kinematic data, developing an ML model to classify patients with nonspecific lower back pain (NSLBP) into high- and low-risk categories. Their models achieved 75% accuracy using a support vector machine (SVM) and 60% accuracy with a multilayer perceptron (MLP). Staartjes et al. [22] assessed the Five-Repetition Sit-to-Stand Test (5R-STS) combined with ML to classify patients with lumbar disk herniation, lumbar spinal stenosis, or NSLBP, achieving approximately 96% accuracy. Liew et al. [23] used motion capture and electromyography to evaluate ML models for classifying low back pain subgroups, achieving an AUC of 90.4% for control vs. current pain and 96.7% for chronic vs. current low back pain. On the same topic, a recent evidence-based investigation identified twenty studies utilizing various AI methodologies, including ML and different DL architectures, to diagnose lumbar degenerative disc disease manifestations such as disc degeneration, herniation, and bulging. Interestingly, the AI models consistently surpassed traditional methods in terms of accuracy, sensitivity, and specificity, with performance metrics ranging from 71.5% to 99% across different diagnostic tasks [24].

NLP is a subfield of AI that addresses interactions between computers and human language. In the area of textual data processing, Ren et al. [25] developed Long Short-Term Memory (LSTM) and extreme gradient boosting (XGBoost) models for distinguishing between lumbar spine stenosis and lumbar disc herniation by analyzing clinical and radiological records from a cohort of 1921 patients. Concerning performance, the LSTM model outperformed the XGBoost model, achieving higher metrics across the board, including an AUC ROC of 0.8487, an accuracy of 0.7818, a recall of 0.9045, an F1 score of 0.8108, and a precision of 0.7347. In contrast, the XGBoost model had lower performance, with an AUC ROC of 0.7565, an accuracy of 0.6961, a recall of 0.7387, an F1 score of 0.7153, and a precision of 0.6934. Similarly, in a prospective observational pilot study, Soin et al. [26] enrolled 246 consecutive patients with spinal pain. Each patient used an iPad to complete a Google form containing 85 specific data points, such as demographic details, pain type, pain score, pain location, pain duration, and functional status scores. These data were then processed by a decision tree ML model that was accurate for pain diagnosis in approximately 72% of the cases.

### 2.4. Large Language Models for Sentiment Analysis

In the context of complex pain conditions like fibromyalgia (FM), which involves altered cognitive and emotional processing, AI can offer valuable insights. Due to their extensive training data, adaptability, contextual understanding, and continual learning, large language models (LLMs) are powerful generative AI instruments [12]. Sentiment analysis using LLMs has been employed to detect subtle nuances in pain expression. Specifically, a study involving 40 patients with FM and 40 controls with chronic pain used the LLM Mistral-7B-Instruct-v0.2 to analyze transcribed responses. The prompt-engineered approach showed superior performance, with an accuracy of 0.87, a precision of 0.92, a recall of 0.84, a specificity of 0.82, and an AUC ROC of 0.86 for distinguishing FM, compared to the ablated approach, which had an accuracy of 0.76 [27]. The statistical significance of these findings suggests that LLM-driven sentiment analysis could enhance FM diagnosis. LLMs are encompassed by the natural language processing methods. It is a field of AI that focuses on the interaction between computers and human language. Therefore, NLP approaches can be used to extract and analyze data from clinical records. Hughes et al. [28] implemented NLP strategies for building and testing a DL model aimed at intercepting pain in the emergency department. 

### 2.5. Neurofunctional Investigations

These techniques offer an in-depth analysis of pain phenomena and neurological processing. For example, Lapitov et al. [29] utilized neuroimaging data, including MRI diffusion tensor and T1-weighted imaging, to identify subtypes of neuropathic facial pain. Using random forest and logistic regression, their ML models achieved up to 95% accuracy in differentiating classical trigeminal neuralgia from healthy controls. Similarly, Peng et al. [30] explored the application of functional near-infrared spectroscopy (fNIRS) for real-time pain detection under general anesthesia, showcasing innovative approaches to pain management. Concerning pain mechanisms, in a study focusing on patients with hypersensitive teeth, researchers used fNIRS to measure hemodynamic cortical responses while patients were in a dental chair. They observed distinct hemodynamic activity in the primary somatosensory cortex (S1) and prefrontal cortex (PFC) triggered by the thermal stimulation of the affected tooth, from the anticipation of pain to its perception. Importantly, the patients’ clinical pain experiences were predicted by their baseline functional connectivity between the S1 and PFC, as well as a specific sequence of hemodynamic responses. This process, involving both sensory-discriminative and cognitive-emotional components, began with activations in the contralateral S1 orofacial region and bilateral PFC during the anticipation of pain (pre-pain phase). These activations were followed by either stable or reduced activity in the PFC and further responses in the S1 when the cold stimuli turned painful (pain phase) [31,32]. Other recent applications of this technique in pain research include studying primary motor area activity in phantom limb phenomena [33], evaluating therapeutic methods for chronic low back pain [34], and assessing pain during acupuncture [35].

## 3. Integration of AI within a Comprehensive Multimodal Model

AI can significantly augment traditional diagnostic methods by adding deeper layers of analysis and insight. Consequently, research has increasingly focused on integrating AI models to analyze complex physiological and clinical data. For instance, Lee et al. [36] conducted a study to develop ML models that objectively classify pain levels using neuroimaging and autonomic metrics. Their study, which involved 53 patients with chronic lower back pain, employed support vector machine (SVM) and support vector regression technologies. The models achieved a classification accuracy of 92.45% and an AUC ROC of 0.97 for pain level detection, with the regression model showing a correlation coefficient (r) of 0.63 in predicting pain in new patients.

In another study, researchers created a database encompassing various biosignals, such as EMG, EDA levels, and ECG. They designed an experimental ML system to classify pain levels based on the biopotential data from 85 participants exposed to controlled heat stimuli. The model, using SVM, achieved a classification accuracy of 91% for differentiating baseline from pain tolerance thresholds and 79% for baseline versus pain threshold [37].

The diagnosis of chronic pain must account for the diverse conditions that shape the pain experience. Soin et al. [26] conducted a pilot study to test ML for diagnosing spinal conditions in chronic pain settings. The study involved 246 patients who provided 85 data points, including demographic and pain-related information, via a Google form on an iPad. A decision tree model processed these data, achieving a 72% accuracy rate compared to practitioner-assigned diagnoses. The study highlighted the potential of AI in enhancing diagnostic accuracy but also emphasized the need for further research to refine these methods and incorporate biopsychosocial factors and data from patient-owned devices.

Cancer pain diagnosis and management often present challenges. A recent study utilized a comprehensive statistical approach, including sensitivity analysis, factorial analysis, and hierarchical clustering on principal components. This analysis integrated demographic, clinical, pain-related variables, and electrodermal activity and ECG signals. The multifactorial analysis revealed links between pain intensity, pain type, Eastern Cooperative Oncology Group (ECOG) performance status, opioid use, and metastasis presence. Clustering analysis identified three distinct patient groups based on pain characteristics, treatments, and ECOG status. Multivariable regression analysis further highlighted associations between pain intensity, breakthrough cancer pain, and opioid dosages [38,39].

## 4. AI-Powered Assessment in Infants and Cognitively Impaired Populations

### 4.1. Pain Diagnosis in Newborn and Infant

The properties of AI systems can be implemented to assess pain levels and guide treatment, particularly in special populations like infants and the elderly, where pain is challenging to evaluate accurately. Infants cannot verbally communicate their pain, often leading to under-recognition and inadequate treatment. This inaccurate pain management in infants is linked to behavioral issues, heightened vigilance, and potential structural brain changes that impact development and learning [40]. To address these challenges, AI techniques analyze behavioral responses such as facial expressions [41], crying sounds [42], and body movements [43], as well as physiological signals like pupil dilation [44], skin conductance [37], heart rate variability [45], and cerebral hemodynamics [46].

Multimodal approaches combine these data sources for more accurate assessments [47]. For example, the PainChek Infant, an mHealth solution, uses AI to evaluate pain intensity based on facial expressions, demonstrating effectiveness in ease of use and accuracy [48]. Similarly, Carlini et al. [49] worked on the UNIFESP [50] and the Classification of Pain Expression (iCOPE) [51] repositories and developed a mobile app utilizing a convolutional neural network (CNN)-based architecture to classify neonate facial expressions as indicative of pain or not, with low latency and offline functionality. Another group of researchers evaluated EGG, an AI-powered interactive toy developed to assess individual pain levels in children. This device engages young patients through an immersive experience incorporating visual, tactile, and auditory stimuli [52]. Additionally, Gholami et al. [53] used ML for evaluating neonate pain intensity through digital imaging analyses.

### 4.2. Cognitively Impaired Elderly Individuals

For cognitively impaired elderly individuals, AI tools analyze nonverbal cues and physiological signals to provide objective pain assessments [54]. The PainChek application is also used for patients with dementia to evaluate pain through facial expressions. For example, a retrospective study by Atee et al. [55] examined the facial expressions in 3144 individuals with dementia using the PainChek Face domain. The study identified facial action units (AUs) as being associated with pain intensity, finding AU7 (eyelid tightening) most prevalent during severe pain. Eye-related AUs were more common at higher pain levels than mouth-related AUs. In another investigation, Babicova et al. [56] tested the tool in UK aged-care residents with advanced dementia. Additionally, video recordings of non-communicative patients during routine activities were analyzed to observe pain behaviors, with ratings performed using the PAINAD score [57]. However, real-world applications of these models have shown mixed results in performance metrics [58].

The main applications are reported in Table 1.

## 5. Limitations, AI Ethics, and Perspectives

While APA methods hold promise for providing more objective assessments of pain intensity, several limitations must be considered. These mostly include a lack of high-quality validation studies, uncertainty regarding which parameters are most appropriate across different clinical settings, and technical challenges such as the timing of their application.

Importantly, the scarcity of diverse, well-constructed behavioral and biosignal-based datasets across different pain conditions presents a significant challenge in ensuring the robustness and generalizability of automated pain assessment systems. Therefore, the development of comprehensive and diverse datasets is a key step in establishing a reliable ground truth, which is necessary for the accurate validation and clinical applicability of these innovative technological solutions.

The research pathway is another crucial issue. A comprehensive approach to pain assessment should integrate both subjective self-reports and objective measures. Additionally, it is essential to use more advanced computational models that account for the variability in clinical data, as current research on objective pain intensity assessment often focuses on point estimation. These point estimates can lead to overconfidence and thus inaccurate predictions, which can be detrimental in clinical settings [59]. Different strategies, such as neural-network-based prediction interval methods, can be adopted to understand the level of uncertainty in pain intensity [60].

Moreover, the integration of AI into clinical workflows requires careful consideration of ethical, legal, and technical factors. Specifically, ethical concerns in AI extend beyond accuracy to issues such as autonomy, data privacy, and transparency. AI systems, even those designed for specific tasks, must be carefully monitored to ensure that they align with human values and ethical standards. This is where the concepts of “human-in-the-loop” (HITL) and algorithmic stewardship come into play. HITL involves integrating human judgment into AI decision-making processes to maintain control and oversight. Meanwhile, algorithmic stewardship focuses on the responsible design, deployment, and monitoring of AI systems to ensure they are ethically designed [61,62]. Moreover, data privacy and security are critical when dealing with AI in healthcare. AI systems rely heavily on patient data, which must be protected to prevent breaches and misuse. Adhering to stringent data protection regulations, such as the White House executive order on the safe, secure, and trustworthy development and use of AI in the United States [63] and the AI Act from the European Parliament [64], is essential for safeguarding sensitive health information. Furthermore, addressing data bias is crucial, as AI models trained on unrepresentative datasets can perpetuate existing inequalities, leading to biased outcomes that affect patient care.

The ethical challenges surrounding AI are further compounded by the needs for transparency and explainability. AI systems, particularly complex models like deep learning, are often seen as “black boxes,” making it difficult to understand how decisions are made. This lack of transparency can undermine trust in AI, especially in high-stakes fields like medicine, where the rationale behind decisions must be clear and understandable. Explainable AI (XAI) seeks to address this by developing methods that make AI decision-making processes more transparent, thereby improving trust and accountability [65,66]. Importantly, XAI is of paramount importance for multimodal approaches that integrate physiological signals, behavioral indicators, and subjective reports. While these strategies may increase the accuracy of AI models in pain assessment, they also present challenges, particularly in terms of interpretability and the “black box” nature of AI.

Additionally, the development of standardized guidelines and protocols for AI implementation will be essential to ensure consistency and safety in patient care [67]. This step is mandatory for integrating AI into routine clinical practice. New professional roles are likely needed to operate at the intersection of technology, philosophy, and policy, working together to shape the guidelines and frameworks that govern AI applications across different domains. Although there is no doubt that pain medicine may undergo significant transformation in this “digital age” [68], the successful adoption of AI in this field will depend on collaborative efforts among researchers, clinicians, and policymakers to create a framework that balances innovation with patient safety.

Despite these limitations, the perspectives are fascinating. For example, AI can be integrated with electronic health record (HER) systems to analyze patient history and current data to provide comprehensive pain assessments. This integration can assist in tracking pain trends over time and support clinical decision making with data-driven insights [69]. Telemedicine is another interesting perspective toward personalized pain management [70]. For example, AI-powered remote monitoring tools can allow patients to record and analyze their clinical conditions and therapy (e.g., analgesic rescue doses) in real time, providing dynamic feedback; additionally, LLM-based virtual assessment tools can analyze video or audio from virtual visits to more accurately assess pain levels and symptoms, and AI-driven chatbots and virtual assistants offer continuous support, answering questions, providing self-management suggestions, and triaging urgent issues. Finally, AI strategies could be implemented to exhaustively address pivotal issues such as opioid prescriptions [71] and misuse [72].

## 6. Conclusions

The potential applications of AI in pain medicine are numerous and intriguing. However, despite its promise, APA methods face important limitations, such as the need for additional validation studies and the difficulty of selecting the most effective parameters for various clinical environments. Reliable data collection is therefore essential for the development of accurate AI models. To address these challenges, it is important to complement traditional clinical evaluations with patient-reported outcomes and biosignal measurements. Given the subjective nature of pain and its complex biopsychosocial dimensions, a multimodal approach that integrates diverse data types within a comprehensive multiprofessional research framework is necessary to obtain more accurate and meaningful insights. Moreover, the integration of AI into pain medicine must be guided by stringent ethical principles. Issues such as data privacy, potential biases in AI algorithms, and the need for transparency in decision-making processes are critical to ensuring that AI applications are effective and equitable.

## Figures and Tables

**Table 1:** Applications of AI for pain diagnosis.

Application	Method, Setting/Aim	Key Findings	Ref.
**AI for Diagnosing Pain Conditions**	ResNet-18 CNN for postoperative pain.	Sensitivity: 89.7%, severe pain detection: 77.5%, pain intensity estimation: 53%	[17]
	Ensemble deep learning model.	Accuracy°: 89%, AUC ROC^: 94% for shoulder pain	[18]
	Transfer learning with pre-trained CNN.	Identified seven-level pain thresholds from facial expressions	[19]
	DarkNet19 pre-trained on ImageNet1K.	Accuracy°: 95% for shoulder pain	[20]
	SVM and ANN. Chronic low back pain.	Classification accuracy°: SVM (75%), ANN (60%) for NSLBP	[21]
	Fuzzy-rule-based system. Low and leg pain.	Accuracy°: 96% using 5R-STS for lumbar conditions	[22]
	Functional data boosting. Low back pain.	AUC^: 90.4% (control vs. current pain), 91.2% (control vs. pain in remission)	[23]
	Different ML and DL architectures (systematic review).	Accuracy°, recall*, and specificity^§^ from 71.5% to 99% in DDD diagnosis	[24]
	LLM (Mistral-7B-Instruct-v0.2) for sentiment analysis in fibromyalgia.	Accuracy°: 87%, precision^‡^: 92%, recall*: 84%, specificity^§^: 82%,	[27]
	LSTM and XGBoost for distinguishing between lumbar spine stenosis and lumbar disc herniation.	LSTM: AUC ROC 0.84, accuracy 0.78, recall 0.90, F1 score of 0.81, and precision of 0.73. XGBoost: AUC ROC 0.75, accuracy 0.69, recall 0.73, F1 score 0.71, and precision 0.69	[25]
**Comprehensive Pain Assessment**	SVM and SVR.	Classification accuracy: 92.45%, AUC^: 0.97	[36]
	Biosignal-based pain recognition. SVM.	Accuracy°: 91% (baseline vs. pain tolerance threshold), 79% (baseline vs. pain threshold)	[37]
	Decision tree ML. Spinal conditions.	Accuracy°: 72%	[26]
	Random forest, logistic regression. Neuropathic facial pain.	Accuracy°: 95%	[29]
**Newborn/Infant**	Facial expression: PCA, LDA, and SVM.	Pain versus non-pain (88.00%), pain versus rest (94.62%), pain versus cry (80.00%), pain versus air puff (83.33%), and pain versus friction (93.00%)	[41]
	Facial expressions and crying	Emotion recognition	[42]
**Elderly/Non-verbal Patients**	Various models and medical device applications (e.g., PainChek)	Enhanced pain assessment	[54,55,56,57,58]

*Legend:* Accuracy°: the proportion of true results (both true positives and true negatives) among the total number of cases examined. It indicates how often the AI model correctly identifies or excludes the condition; AUC ROC^: represents the model’s ability to distinguish between classes, with values closer to 1 indicating better performance. A higher AUC ROC means the model is better at distinguishing between those with and without the condition; Precision^‡^ (or positive predictive value): the proportion of true positives among the total number of cases that the model predicted as positive. It reflects the accuracy of the model in predicting positive instances; Recall* (or sensitivity or true positive rate): the proportion of actual positives that are correctly identified by the model. It indicates how well the model can identify positive instances; Specificity^§^: the proportion of true negatives that are correctly identified by the model. It reflects the model’s ability to correctly exclude individuals who do not have the condition. *Abbreviations*: CNN, convolutional neural network; AUC, area under the curve; ROC, receiver operating characteristic; SVM, support vector machine; ANN, artificial neural network; NSLBP, nonspecific low back pain; 5R-STS, Five-Repetition Sit-to-Stand Test; DDD, degenerative disc disease; LSTM, Long Short-Term Memory; XGBoost, extreme gradient boosting; LLM, large language model; SVR, support vector regression; PCA, principal component analysis; LDA, linear discriminant analysis.

## Data Availability

Data collected for the review are available from the corresponding author upon reasonable request.
